# Evaluation of contrast sensitivity in non-high-risk proliferative diabetic retinopathy treated with panretinal photocoagulation with and without intravitreal injections of ranibizumab

**DOI:** 10.5935/0004-2749.20220006

**Published:** 2022

**Authors:** Zubir S. Rentiya, Daniel A. Ferraz, Robert Hutnik, Junun Bae, Cleide G. Machado, Cristina Mucciolli, Augusto Alves L. da Motta, Lucas Z. Ribeiro, Zeyu Guan, Rony Carlos Preti, Walter Y. Takahashi

**Affiliations:** 1 Johns Hopkins University School of Medicine, Department of Radiology, Neurology, Ophthalmology, Baltimore, MD, USA; 2 Division of Ophthalmology, University of São Paulo, São Paulo, SP, Brazil; 3 Division of Ophthalmology, Federal University of São Paulo, São Paulo, SP,Brazil; 4 Moorfield’s Eye Hospital, London, UK; 5 Stony Brook Medicine, Department of Surgery, Stony Brook, NY, USA; 6 Lake Erie College of Osteopathic Medicine, Erie, PA, USA

**Keywords:** Diabetic retinopathy, Light coagulation, Ranibizumab, Bevacizumab, Contrast sensitivity, Vascular endothelial growth factor A, Intravitreal injection, Retinopatia diabética, Fotocoagulação, Ranibizumab, Bevacizumab, Sensibilidade de contraste, Fator A de crescimento do endotélio vascular, Injeção intravítrea

## Abstract

**Purpose:**

To evaluate contrast sensitivity in non-high-risk, treatment-naïve proliferative diabetic retinopathy patients treated with panretinal photocoagulation and intravitreal injections of ranibizumab) versus panretinal photocoagulation alone.

**Methods:**

Sixty eyes of 30 patients with bilateral proliferative diabetic retinopathy were randomized into two groups: one received panretinal photocoagulation and ranibizumab injections (study group), while the other received panretinal photocoagulation alone (control group). All eyes were treated with panretinal photocoagulation in three sessions according to the Early Treatment Diabetic Retinopathy Study guidelines. Contrast sensitivity measurements were performed under photopic conditions (85 cd/m^2^) with the Visual Contrast Test Sensitivity 6500 chart, allowing for the evaluation of five spatial frequencies with sine wave grating charts: 1.5, 3.0, 6.0, 12.0, and 18.0 cycles per degree (cpd). Outcomes were measured in contrast sensitivity threshold scores among and within groups, from baseline to 1, 3, and 6 months.

**Results:**

Fifty-eight eyes (28 in the study group and 30 in the control group) reached the study endpoint. A comparative analysis of changes in contrast sensitivity between the groups showed significant differences mainly in low frequencies as follows: at month 1 in 1.5 cpd (p=0.001) and 3.0 cpd (p=0.04); at month 3 in 1.5 cpd (p=0.016), and at month 6 in 1.5 cpd (p=0.001) and 3.0 cpd (p=0.026) in favor of the study group.

**Conclusions:**

In eyes of patients with non-high-risk proliferative diabetic retinopathy, panretinal photocoagulation treatment with ranibizumab appears to cause less damage to contrast sensitivity compared with panretinal photocoagulation treatment alone. Thus, our evaluation of contrast sensitivity may support the use of ranabizumab as an adjuvant to panretinal photocoagulation for the treatment of proliferative diabetic retinopathy.

## Abstract (pt)

**Objetivos:**

Avaliar a sensibilidade ao contraste em pacientes virgens de tratamento com retinopatia diabética proliferativa de não alto risco, submetidos a panfotocoagulação retiniana com injeções intravítreas de ranibizumabe versus pan fotocoagulação isolada.

**Métodos:**

Sessenta olhos de 30 pa cientes foram randomizados em dois grupos: um submetido a panfotocoagulação com injeções de ranibizumabe (grupo estudo), e o outro submetimedo a panfotocoagulação isolada (grupo controle). Todos olhos foram tratados em 3 sessões de laser, se guindo recomendação do *Early Treatment Diabetic Retinopathy Study* (ETDRS). Avaliação da sensibilidade ao contraste foi realizada sob condições fotópicas (85 cd/m2) com tabela *Visual Contrast Test Sensitivity* 6500, permitindo avaliação de cinco frequências espaciais medidas com redes senoidais: 1.5, 3.0, 6.0, 12.0 e 18.0 ciclos por grau de ângulo visual (cpd). Foram realizadas medidas dos limiares de sensibilidade ao contraste intra e entre grupos na visita inicial, no 1º, 3º, e 6º mês de seguimento.

**Resultados:**

Cinquenta e oito olhos, 28 do grupo estudo e 30 do grupo controle, atingiram o término do estudo. Análise comparativa da SC entre os grupos mostrou diferença estatisticamente significante, nas baixas frequências espaciais, no 1º mês em 1.5 cpd (p=0,001) e 3.0 cpd (p=0,04), no 3º mês em 1.5 cpd (p=0,016) e no 6º mês em 3.0 cpd (p=0,026) a favor do grupo estudo.

**Conclusão:**

O tratamento com panfotocoagulação associada a injeção de ranibizumabe parece causar menos danos a sensibilidade ao contraste quando comparada com panfotocoagulação isolada em olhos com retinopatia diabética proliferativa de não alto risco. Dessa forma, os resultados apresentados podem justificar a associação do ranibizumabe à panfotocoagulação nestes pacientes.

## INTRODUCTION

Proliferative diabetic retinopathy (PDR) and diabetic macular edema (DME) are the main causes of severe and moderate visual loss in patients with diabetes^(1)^. Vascular endothelial growth factor (VEGF) is the principal cytokine related to the development of these conditions^(2)^.

Panretinal laser photocoagulation (PRP) is considered the standard treatment of PDR according to The Diabetic Retinopathy Study Research Group and supported by the Early Treatment Diabetic Retinopathy Study (ETDRS)^(3-5)^. Nevertheless, in approximately one-quarter of treated eyes, new vessels continue to grow despite the PRP or recur after partial or complete initial regression, requiring enhanced PRP. PRP may also affect visual function, visual acuity (VA), and contrast sensitivity (CS), leading to macular edema caused by the laser itself^(6)^. Although the long-term visual prognosis of patients with PDR treated with PRP is generally good^(7)^, progression of visual loss continues to occur in nearly 5% of patients despite treatment^(4)^.

CS and VA are important independent factors in explanatory models of visual disability^(8)^. VA provides an accurate measure of the patient’s ability to resolve detail at high contrast, while CS describes the ability to see low-contrast patterns. The VA test is the most common test used to measure the visual processing system using spatial resolution. It evaluates the optotypes with a higher degree of contrast. However, real-world objects have different degrees of variability in contrast and spatial frequency^(9,10)^.

While measurement of VA alone is useful, it does not address the loss of CS, which is a frequent consequence of retinal diseases and can have a serious impact on the quality of life and functional ability of patients^(11)^. Therefore, CS testing provides additional measurement of a patient’s ability to see low-contrast patterns and information on one’s visual function than VA alone^(9,10)^.

Ranibizumab (RBZ) is a humanized monoclonal antibody fragment which binds to multiple variants of the VEGF-A^(12)^, leading to regression of macular edema and retinal neovessels from DR, thereby improving DME and PDR, respectively. Recently, Ferraz et al.^(13)^, investigated the use of a regimen of two RBZ injections to augment PRP in patients with non-high-risk PDR. They found that RBZ had a protective effect against VA loss compared with PRP alone.

Several studies have compared the effects of PRP treat ment alone versus PRP combined with RBZ injection on VA; however, none thus far estimated the effects of treatment on CS. In this study, we investigated whether the use of RBZ injections as an adjuvant to PRP reduces the negative impact of PRP alone on CS in treatment-naïve eyes with non-high-risk PDR.

## METHODS

### Patient population

This interventional, prospective, blinded, and randomized study was conducted between July 2011 and June 2012 at the Retina Service of the Division of Ophthalmology, University of São Paulo (São Paulo, Brazil). The study was approved by the institutional review board and ethics committee, and conducted in accordance with the principles of the Declaration of Helsinki. Written informed consent was provided by all patients. This study is listed under ClincalTrials.gov Identifier NCT01746563.

The inclusion criteria for this study were patients with type II diabetes mellitus, aged ≥18 years, with all of the following criteria: 1) non-high-risk PDR in both eyes according to the ETDRS Diabetic Retinopathy Severity Score levels 61 (mild PDR) and 65 (moderate PDR); 2) ETDRS letters-measured best-corrected VA better than 20/60 on Snellen equivalent; and 3) no prior treatment of DR (of any type) in either eye^(14)^.

The exclusion criteria were: 1) aphakia; 2) macular ischemia; 3) cataract surgery in the past 12 months; 4) history of glaucoma or ocular hypertension; 5) loss of vision as a result of other causes; 6) history of systemic corticosteroid therapy within the last 3 months; 7) severe systemic disease other than diabetes mellitus; and 8) any condition that could affect follow-up or documentation, including pre-retinal or vitreous hemorrhage (VH).

During the enrollment period, 450 patients with DR were evaluated for this study, and a total of 30 patients (60 eyes) met the eligibility criteria. These eyes were randomized into the study group (SG) and control group (CG) by means of an in-house, biostatistician-designed randomization computer program. From the 60 eyes, 58 eyes (28 in the SG and 30 in the CG) completed the study. Two eyes from the SG were excluded due to VH prior to RBZ treatment but after the screening visit, which did not resolve and required vitrectomy several weeks later; these were not included in the analysis for CS.

All participants underwent an ophthalmologic examination consisting of best-corrected VA using the Snellen chart at 4 m, anterior segment slit-lamp examination, intraocular pressure measured by Goldmann applanation tonometry, and dilated retinal examination with a 78-diopter lens.

A single certified examiner performed CS measurements using the Visual Contrast Test Sensitivity 6500 protocol. Digital color fundus photography and fluorescein angiography (FA) were obtained using a 30° fundus camera system (TRC-50X/IMAGEnet; Topcon, Tokyo, Japan). We used the EDTRS seven-field protocol for the acquisition of fundus images, capturing images of the posterior pole and the four peripheral quadrants to detect some degree of retinal ischemia using the FA.

The Fast Macular Scan Protocol (six linear 6-mm scans oriented at intervals of 30° centered at the fovea) was performed using a third-generation time-domain optical coherence tomography (OCT) device (Stratus; Model 3000; Carl Zeiss Ophthalmic Systems Inc; Humphrey Division, Dublin, CA, USA). Spectral domain-OCT has been the main OCT device used worldwide since 2006, and macular thicknesses measured with the two devices were well correlated (r=0.977) in cases of DME^(15)^.

Central subfield thickness was automatically generated by the incorporated Stratus OCT software, which calculates the distance between the internal limiting membrane and the retinal pigment epithelium. The central subfield thickness was subsequently obtained from the 1,000-µm diameter ETDRS inner circle grid map placed over the macula. Values ≥250 µm were indicative of DME^(15)^. All examiners were blinded throughout the study period. The patients were classified by the severity of DR according to the ETDRS Diabetic Retinopathy Severity Score, in which non-high-risk PDR includes level 61 (new vessels elsewhere, <0.5 disc area in one or more quadrants), and level 65 (new vessels elsewhere, ≥0.5 disc area in one or more quadrants; or new vessels of the disc, <1/4-1/3 disc area)^(14)^.

### Intervention methodology

The baseline visit occurred within 10 days after the initial screenings. At baseline, the eyes of each patient were randomly assigned to receive 0.5 mg RBZ injection (SG) and sham injection in the other eye (CG). Baseline CS measurements for both groups were performed prior to injection. One week later, both eyes received bilateral full scatter PRP treatment, performed in three weekly sessions according to the ETDRS guidelines^(4)^. RBZ (0.5 mg) or sham injections were re-administered at month 1 according to the randomization. All intravitreal injections were performed under sterile conditions using topical anesthesia followed by 0.3% ciprofloxacin eye drops four times daily for 5 days^(14)^. A single retina specialist (RCP) performed all laser procedures, while another retina specialist (DAF) performed all injections.

The follow-up examinations at 1, 3, and 6 months after treatment included detailed ophthalmologic examinations (applanation tonometry, non-dilated and dilated slit-lamp biomicroscopic examination, indirect fundus examination, CS measurements, fundus photography, FA, and OCT).

### Statistical analysis

The collected data were analyzed using descriptive statistics. A non-normal distribution was found using the Shapiro-Wilk W-test. Baseline characteristics were assessed using either the independent sample t-test or Mann-Whitney *U* test for continuous variables, and either the chi-squared test or Fisher’s exact test for categorical variables.

The Wilcoxon signed-rank test was utilized for intergroup and intragroup comparisons of CS in eyes with and without DME, followed by a pre-planned sub-group analysis for eyes with and without DME. The Mann-Whitney *U* test was used for intergroup and inter-subgroup comparisons. SPSS 15.0 for Windows (SPSS, Inc., Chicago, IL, USA) software was used for all statistical analyses. The null hypothesis was rejected for p-values ≤0.05.

## RESULTS

For the 30 patients enrolled, the mean ± standard deviation (SD) age and time from diabetes mellitus diagnosis were 52.3 ± 7.8 years and 14 ± 6.4 years, respectively. Mean ± SD glycated hemoglobin (HbA1C) level was 8.8 ± 1.1%. Patient demography and ocular characteristics are demonstrated in Table 1.

**Table 1:** Baseline Demographics and Ocular Characteristics of All Patients

Parameter	All patients n=30		DME			No DME		
			Control group (n=14)	RBZ group (n=15)	p-value	Control group (n=16)	RBZ group (n=15)	p-value
Mean age, years (SD),	52.6 (7.9)		50.8 (7.0)	53.2 (7.7)	0.38	53.1 (8.7)	51.9 (8.2)	0.69
Sex (female), n (%)	15 (53.6)		7 (50.0)	9 (60.0)	0.72	8 (50.0)	6 (46.2)	1.00
Race, n (%)					0.71			0.88
Caucasian	12 (42.9)		7 (50.0)	9 (60.0)		5 (31.3)	3 (23.1)	
African American	14 (50.0)		7 (50.0)	6 (40.0)		9 (56.3)	8 (61.5)	
Asian	2 (7.1)		0 (0)	0 (0)		2 (12.5)	2 (15.4)	
Mean duration of diabetes, years (SD)	14 (6.5)		12.4 (7.2)	13.6 (7.8)	0.71	15.4(5.7)	14.5 (4.8)	0.59
Mean HbA1c, % (SD)	8.9 (1.1)		9.0 (1.2)	8.8 (1.1)	0.79	8.7 (1.0)	8.9 (1.0)	0.62
Insulin users, n (%)	19 (67.9)		9 (64.3)	11 (73.3)	0.7	12 (75.0)	8 (61.5)	0.68
Hypertension, n (%)	25 (89.3)		12 (85.7)	13 (86.7)	1.00	15 (93.8)	12 (92.3)	1.00
Hypercholesterolemia, n (%)	11 (39.3)		5 (35.7)	6 (40.0)	1.00	7 (43.8)	5 (38.5)	1.00
**Ocular characteristics**	**Control group (=30)**	**RBZ group (n=28)**	**p-value**	**Control group (n=14)**	**RBZ group (n=15)**	**Control group p-value (n=16)**	**RBZ group (n=13)**	**p-value**
Mean BCVA (SD) ETDRS at 4 m	44.4 (13.8)	44.8 (12.8) 0.92		41.8 (17.5)	42.1 (16.1)	0.75 46.8 (9.5)	47.9 (6.6)	0.71
Mean intraocular pressure (mmHg)	14.1 (2.2)	13.9 (2.1)	0.72	14.8 (2.3)	14.3 (2.3)	0.60 13.5 (2.0)	13.4 (2.0)	0.88
Phakic, n (%)	23 (76.7)	19 (67.9)	0.56	11 (78.6)	10 (66.7)	0.68 12 (75.0)	9 (69.2)	1.00

BCVA= best-corrected visual acuity; DME= diabetic macular edema; ETDRS= early treatment diabetic retinopathy study; Hb= hemoglobin; RBZ= ranibizumab; SD= standard deviation.

**Figure 1:**
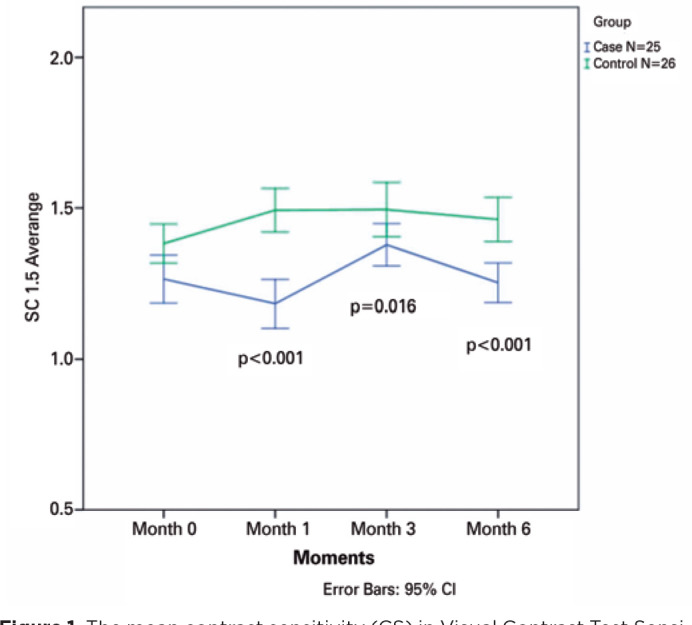
The mean contrast sensitivity (CS) in Visual Contrast Test Sensitivity grade at 1.5 cycles read at 4 m is plotted for all visits from baseline to month 6 for all patients in the ranibizumab (RBZ) and control groups. CI, confidence interval.

**Figure 2:**
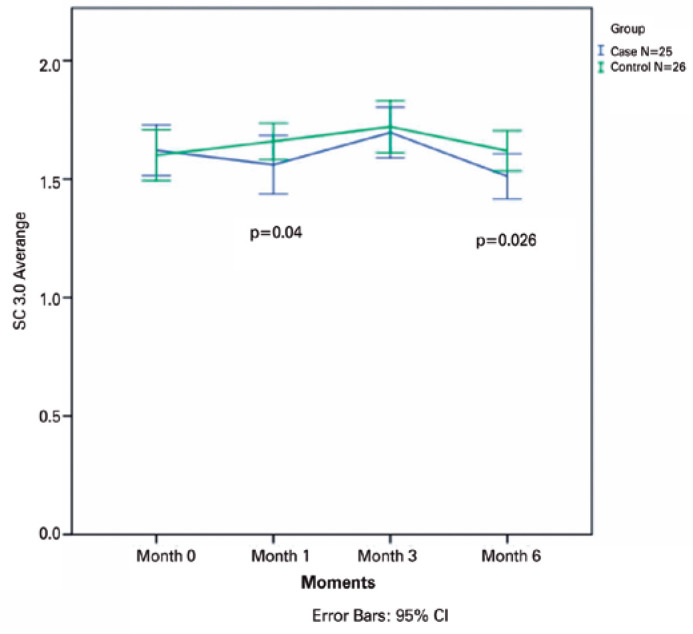
The mean contrast sensitivity (CS) in Visual Contrast Test Sensitivity grade at 3.0 cycles read at 4 m is plotted for all visits from baseline to month 6 for all patients in the ranibizumab (RBZ) and control groups. CI, confidence interval.

**Figure 3:**
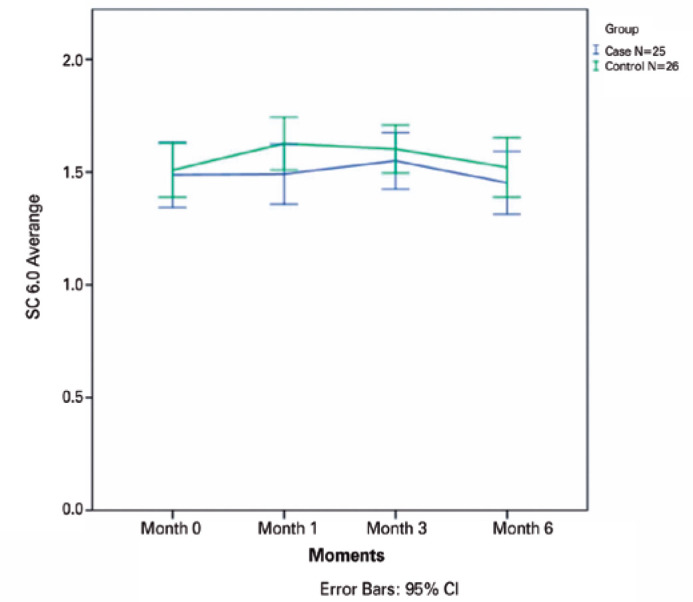
The mean contrast sensitivity (CS) in Visual Contrast Test Sensitivity grade at 6.0 cycles read at 4 m is plotted for all visits from baseline to month 6 for all patients in the ranibizumab (RBZ) and control groups. CI, confidence interval.

**Figure 4:**
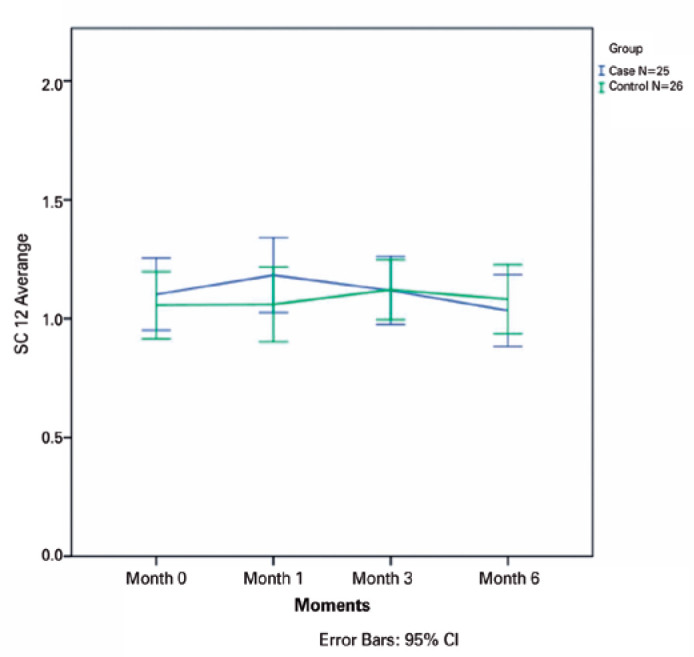
The mean contrast sensitivity (CS) in Visual Contrast Test Sensitivity grade at 12.0 cycles read at 4 m is plotted for all visits from baseline to month 6 for all patients in the ranibizumab (RBZ) and control groups. CI, confidence interval.

**Figure 5:**
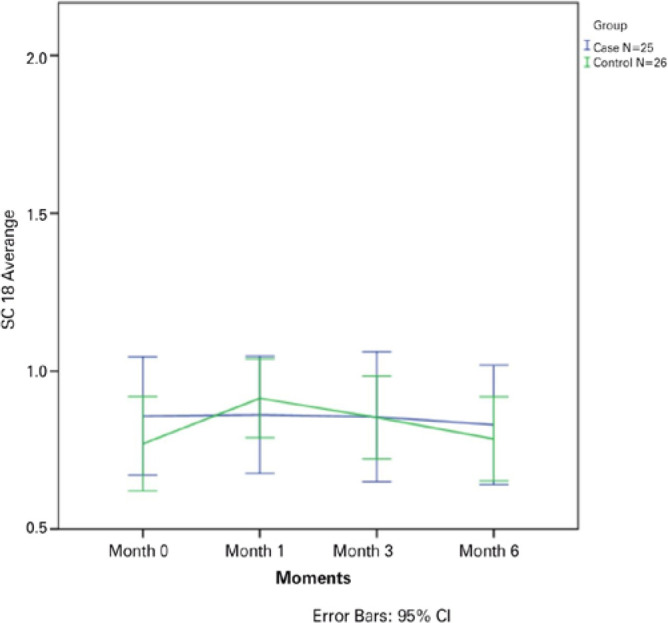
The mean contrast sensitivity (CS) in Visual Contrast Test Sensitivity grade at 18.0 cycles read at 4 m is plotted for all visits from baseline to month 6 for all patients in the ranibizumab (RBZ) and control groups. CI, confidence interval.

The subgroups (patients with and without DME) were well balanced in terms of demographics and ocular characteristics at baseline. Details of the baseline demographics are presented in table 1. A comparative analysis of CS in all eyes did not show a significant difference between the two groups at baseline in 1.5, 3, 6, 12, or 18 cycles per degree (cpd) (p=0.68, p=0.70, p=0.33, p=0.30, and p=0.90, respectively) (Figures 1-5).

The baseline CG and SG included 30 eyes each, receiving a mean ± SD of 1,466.9 ± 202.6 spots for PRP. There was no statistically significant difference between the mean number of burns given in each group or among subgroups (patients with and without DME). Focal laser or additional PRP were allowed at month 3, when 33% and 32.1% of the patients in the CG and SG, respectively, required additional PRP. At 3-month follow-up, focal laser was performed in 63% and 53.6% of the patients, respectively. There was no significant difference in the proportion of eyes that received additional PRP or focal laser treatment in the two groups (p=1.00 and 0.60, respectively) (Tables 2 and 3).

**Table 2:** Summary of Laser Treatments in All Patients

Laser treatment	Control group (n=30)	RBZ group (n=28)	p-value
Mean number of spots for PRP (SD)	1,460.8 (236.6)	1,473.4 (162.6)	0.82
Patients receiving additional PRP at month 3, n (%)	10 (33.3)	9 (32.1)	1.00
Mean number of spots for additional PRP at month 3, n (SD)	334.8 (159.9)	230.4 (79.4)	0.10
Patients receiving additional focal laser at month 3, n (%)	19 (63.3)	15 (53.6)	0.60
Mean number of spots for focal laser at month 3, n (SD)	110.1 (21.8)	110.9 (28.1)	0.93

PRP= panretinal photocoagulation; RBZ= ranibizumab; SD= standard deviation.

**Table 3:** Summary of Laser Treatments in Patients with and without Diabetic Macular Edema

Laser treatment	DME			No DME		
	Control group (n=14)	RBZ group (n=15)	p-value	Control group (n=16)	RBZ group (n=13)	p-value
Mean number of spots for PRP, n (SD)	1,421.6 (315.6)	1,459.4 (180.8)	0.69	1,495.1 (138.5)	1,489.5 (144.4)	0.92
Patients receiving additional PRP at month 3, n (%)	4 (28.6)	4 (26.7)	1.00	6 (36.5)	5 (38.5)	1.00
Mean number of spots for additional PRP at month 3, n (SD)	254.0 (37.3)	201.8 (68.8)	0.23	388.7 (191.0)	253.4 (104.7)	0.19
Patients receiving additional focal laser at month 3, n (%)	12 (85.7)	9 (60.0)	0.22	7 (43.8)	6 (46.2)	1.00
Mean number of spots for focal laser at month 3, n (SD)	104.2 (16.8)	106.2 (31.0)	0.85	120.3 (26.7)	118.0 (24.0)	0.88

DME= diabetic macular edema; PRP= panretinal photocoagulation; RBZ= ranibizumab; SD= standard deviation.

### Primary outcomes

All the results were expressed in logMAR. Pretreatment, there was no difference of CS in all spatial fre quencies in the SG and CG (p>0.05). However, at 1 month, log CS was decreased in SG by 0.082 in 1.5 cpd and 0.061 in 3 cpd, increased by 0.003 in 6 cpd, decreased by 0.027 in 12 cpd, and increased by 0.004 in 18 cpd. In the CG, we observed increases by 0.011 in 1.5 cpd, 0.059 in 3 cpd, 0.118 in 6 cpd, 0.115 in 12 cpd and 0.144 in 18 cpd.

Those changes were significant at 1.5 cpd (p=0.004) in the SG and at 1.5 cpd (p=0.017), 6 cpd (p=0.007), and 18 cpd (p=0.001) in the CG. A comparative analysis of changes in CS between the groups showed significant differences at month 1 in 1.5 (p=0.000) and 3.0 cpd (p=0.04), in favor of the SG.

At month 3, CS was increased in the SG by 0.114 in 1.5 cpd, 0.075 in 3 cpd, 0.062 in 6 cpd, 0.016 in 12 cpd, and a decrease by 0.003 in 18 cpd. In the CG, there was an increase by 0.113 in 1.5 cpd, 0.120 in 3 cpd, 0.094 in 6 cpd, 0.065 in 12 cpd, and 0.083 in 18 cpd. These changes were significant at 1.5 cpd (p=0.036) in the SG and at 1.5 cpd (p=0.024), 6 cpd (p=0.022), and 18 cpd (p=0.038) in the CG. A comparative analysis of changes in CS between the groups showed significant differences at month 3 in 1.5 cpd (p=0.016).

At month 6, CS was decreased in the SG by 0.012 in 1.5 cpd, 0.109 in 3 cpd, 0.036 in 6 cpd, 0.068 in 12 cpd, and 0.028 in 18 cpd. In contrast, the CS in the CG increased by 0.080 in 1.5 cpd, 0.019 in 3 cpd, 0.012 in 6 cpd, 0.025 in 12 cpd, and 0.023 in 18 cpd. Those changes were statistically significant only at 3.0 cpd (p=0.023) in the SG. A comparative analysis of changes in CS between the groups showed significant differences at month 6 in 1.5 cpd (p=0.001) and 3.0 cpd (p=0.026) (Figures 1-5).

### Secondary outcomes

#### Patients with DME

Among the 29 eyes with DME (15 in the SG and 14 in the CG), at 1 month, CS was decreased in the SG by 0.115 in 1.5 cpd and 0.113 in 3 cpd, increased by 0.015 in 6 cpd, decreased by 0.062 in 12 cpd, and increased by 0.067 in 18 cpd. In the CG, there was an increase by 0.021 in 1.5 cpd, a decrease by 0.015 in 3 cpd, an increase by 0.092 in 6 cpd, a decrease by 0.009 in 12 cpd, and an increase by 0.082 in 18 cpd. Those changes were significant at 1.5 cpd (p=0.036) in the SG and at 1.5 cpd (p=0.024), 6 cpd (p=0.022), and 18 cpd (p=0.038) in the CG. A comparative analysis of changes in CS between the groups showed significant differences at month 1 in 1.5 cpd (p=0.001).

At month 3, CS was increased in the SG by 0.115 in 1.5 cpd, 0.115 in 3 cpd, and 0.050 in 6 cpd, and decreased by 0.001 in 12 cpd and 0.018 in 18 cpd. In the CG, there was an increase by 0.150 in 1.5 cpd, 0.149 in 3 cpd, 0.078 in 6 cpd, 0.015 in 12 cpd, and 0.008 in 18 cpd. Those changes were significant at 1.5 cpd (p=0.036) in the SG and at 1.5 cpd (p=0.024), 6 cpd (p=0.022), and 18 cpd (p=0.038) in the CG. A comparative analysis of changes in CS between the groups showed significant differences at month 3 in 1.5 cpd (p=0.016).

At month 6, CS was decreased in the SG by 0.041 in 1.5 cpd, decreased by 0.061 in 3 cpd, increased 0.015 in 6 cpd, decreased by 0.062 in 12 cpd, and increased by 0.067 in 18 cpd. In contrast, CS in the CG increased by 0.027 in 1.5 cpd, and decreased by 0.007 in 3 cpd, 0.007 in 6 cpd, 0.021 in 12 cpd, and 0.011, and decreased by 0.007 in 18 cpd. Those changes were significant at 1.5 cpd (p=0.036) in the SG and at 1.5 (3 cpd) cpd (p=0.024), 6 cpd (p=0.022), and 18 cpd (p=0.038) in the CG. A comparative analysis of changes in CS between the groups showed statistically significant differences at month 6 in 1.5 cpd (p=0.001).

### Patients without DME

In 29 eyes without DME (13 in the SG and 16 in the CG), at 1 month, log CS was decreased in the SG by 0.053 in 1.5 cpd, 0.016 in 3 cpd, and 0.007 in 6 cpd, increased by 0.003 in 12 cpd, and decreased by 0.050 in 18 cpd. In the CG, there was an increase by 0.080 in 1.5 cpd, 0.063 in 3 cpd, 0.108 in 6 cpd, 0.155 in 12 cpd, and 0.185 in 18 cpd. A comparative analysis of changes in CS between the groups did not show statistically significant differences at 1 month.

At month 3, CS was increased in the SG by 0.113 in 1.5 cpd, 0.042 in 3 cpd, 0.073 in 6 cpd, 0.031 in 12 cpd, and 0.011 in 18 cpd. In the CG, there was an increase by 0.017 in 1.5 cpd, 0.111 in 3 cpd, 0.120 in 6 cpd, 0.062 in 12 cpd, and 0.012 in 18 cpd. A comparative analysis of changes in CS between the groups did not show statistically significant differences at month 3.

At month 6, CS was increased in the SG by 0.012 in 1.5 cpd, and decreased by 0.151 in 3 cpd, 0.025 in 6 cpd, 0.092 in 12 cpd, and 0.037 in 18 cpd. In contrast, the CS in the CG increased by 0.065 in 1.5 cpd, 0.028 in 3 cpd, 0.013 in 6 cpd, 0.038 in 12 cpd, and 0.027 in 18 cpd. A comparative analysis of changes in CS between the groups showed statistically significant differences at month 6 in 18 cpd (p<0.05).

### VH

Eight of 30 patients (26.7%) in the CG and four of 30 patients (13.3%) in the SG developed VH (p=0.33). Two patients in the SG developed VH prior to RBZ injection and underwent vitrectomy and endolaser treatment.

There was no cataract surgery performed or a significant increase in mean IOP observed during the study period.

The intravitreal injection procedure was well tolerated. There was no clinical evidence of sterile inflammation, endophthalmitis, or ocular toxicity. Moreover, there were no serious drug-related adverse events in the 28 eyes that received RBZ.

## DISCUSSION

Previous studies reported that 25-43% of patients with PDR treated by PRP alone may develop an increase in macular thickness and worsening of their macular edema, resulting in visual disturbances (e.g., CS deterioration)^(15-18)^. The role of the protective adjunctive effect of RBZ in the stabilization of CS has yet to be elucidated. In the present study, we evaluated the possible adjunctive effects of two RBZ injections in combination with laser photocoagulation for the management of patients with treatment-naïve non-high-risk PDR with or without DME. In addition, we examined the mechanism through which this adjuvant treatment can affect the CS.

The exact reason for the impairment of CS after PRP remains unclear, and the debate continues. Mackie et al.^(19)^ have demonstrated impairment in the CS after treatment with PRP. Similar findings were reported by Khosla et al.^(20)^; impairment in the CS after treatment with PRP was observed at the initial follow-up and was not maintained at the end of 3 months. However, Canning et al.^(21)^ showed some conflicting results. In the present study, we evaluated eyes with non-high-risk PDR. The results of a 6-month analysis showed that eyes treated with RBZ and PRP did not have an impairment of CS at the end of 3 and 6 months compared with placebo (PRP and sham injections).

Preti et al.^(22)^ treated high-risk PDR eyes with either bevacizumab (BVZ) or sham injections at baseline, followed by grid laser and PRP within 14 days and another BVZ or sham injection 1 month later. At the 6-month endpoint, they reported a decrease in CS in the sham-treated patients compared with BVZ-treated patients; however, the difference was not statistically significant.

Our results showed worsening of CS in most spatial frequencies in the CG compared with that of pre-treatment eyes. These changes were maintained throughout the entire follow-up with significant results (at 1.5 cpd, 6.0 cpd, and 18.0 cpd at months 1 and 3). From these results, we noted a trend of improvement in CS in the SG during the follow-up. The CS did not worsen in most frequencies in the SG, and there were improvements in some of the frequencies at 1.5 cpd at months 1 and 3, and 3.0 cpd at month 3. Thus, our results strongly suggest that RBZ is associated with some protection of CS after PRP. This protection reaches a plateau at month 1, most likely as a result of the timing of the administration of the two RBZ injections at baseline and month 1. Subsequently, a trend showing deterioration of CS after the PRP was observed.

Our data show deterioration in all spatial frequencies in all stages of the follow-up. This is in agreement with previous studies that indicated a deleterious effect of PRP on CS function^(20-22)^. Decreased CS, especially in mid-spatial frequencies, may explain why some patients experience difficulties in daily life, such as with facial recognition in low contrast. RBZ was strongly involved in the maintenance of CS in patients treated with PRP. In fact, the SG reached a better CS on month 6 at 1.5 and 3.0 cpd (p<0.05); this finding supports our hypothesis. Even with the worsening of CS during the follow-up after a decrease in the concentration of RBZ in the vitreous, the end points at month 6 showed better results in the SG.

As the human vision gathers information from all ranges of contrasts and frequencies, there is no single test able to assess all aspects of the daily visual performance. Since CS was first defined by Schade in 1956, it has been widely accepted as a mode of visual functional assessment alongside VA assessment^(23)^. CS testing provides a more comprehensive assessment of the functional vision in comparison with the Snellen VA assessment. Even in low spatial frequencies, it also provides a good assessment of the functionality of the vision. This translates into being able to carry out daily tasks, including night driving, mobility, reading speed, computer task accuracy, and watching television. This study showed that RBZ therapy as an adjuvant to PRP reduced the risk of CS and VA loss. Therefore, we believe that patients who received RBZ therapy will be able to maintain superior functional vision in comparison with those who did not receive RBZ injections. Of note, the patients with DME benefitted more than those without DME. The action of RBZ in reducing DME and preventing its development after the PRP could explain why these patients exhibited better CS results.

In the present study, the central subfield thickness measurements were performed with Stratus time-domain OCT. The data were highly correlated to those obtained from the spectral domain OCT, but differed by almost 50 µm due to different segmentations in each machine^(15,24)^.

Importantly, 20% of the patients developed some degree of VH. These patients developed VH between the screening visit in the clinic and the baseline visit (scheduled within 10 days from the clinic visit) that did not allow the investigator to provide RBZ treatment. The considerable prevalence of VH during the follow-up period in both groups could be reduced if the screening of the patients was performed with indirect ophthalmoscopy or wide-field FA. Through this approach, neovascularization elsewhere and its extension in the retinal periphery could have been more precisely diagnosed.

It would be reasonable to acknowledge that the sample size of this study was small and the follow-up period of 6 months may not have been sufficient to determine the effects and prognosis of the treatment 24-36 months later. However, this study is also characterized by strengths rendering it reliable. For example, the same experienced retina specialist performed the PRP treatments for both the control and testing groups. This suggests that all patients in this study had similar baseline PRP procedures, number of laser burns, and number of sessions attended. Another strength of this study was that we were able to randomly assign each eye of the same patient into the treatment versus non-treatment group, given that both eyes of the patient had similar baseline characteristics. This allowed us to compare between the two treatment groups, eliminating other environmental factors that could have led to confounding effects in the results. In this study, we included patients with PDR (in conjunction with or without DME) who had not received any previous treatment. This is a common scenario encountered in developing countries. We believe that, although the recruitment of patients with PDR treatment-naïve eyes may be viewed as a challenge and a study limitation, this study provided a good treatment guideline to improve the visual functionalities of patients receiving this treatment, which, as mentioned before, is prevalent in developing countries.

In conclusion, an investigation with a larger sample size and a longer follow-up period should be conducted to further strengthen the results of this study. Nevertheless, this analysis has shown that the administration of intravitreal RBZ injections alongside PRP is effective in reducing CS loss in treatment-naïve eyes with non-high-risk PDR. We believe that this study provides evidence for the use of RBZ injections, both before and after PRP treatment in patients with DME and non-high-risk PDR.
